# Kawasaki Disease and Inborn Errors of Immunity: Exploring the Link and Implications

**DOI:** 10.3390/diagnostics13132151

**Published:** 2023-06-23

**Authors:** Saniya Sharma, Pallavi L Nadig, Rakesh Kumar Pilania, Kaushal Sharma, Manpreet Dhaliwal, Amit Rawat, Surjit Singh

**Affiliations:** Allergy Immunology Unit, Department of Pediatrics, Postgraduate Institute of Medical Education and Research, Chandigarh 160012, India; drsaniya.sharma@gmail.com (S.S.); poorvinadig91@gmail.com (P.L.N.); kksukv@gmail.com (K.S.); manpreet326@gmail.com (M.D.); rawatamit@yahoo.com (A.R.); surjitsinghpgi@rediffmail.com (S.S.)

**Keywords:** inborn errors of immunity, Kawasaki disease, primary immunodeficiency diseases, pediatric vasculitis

## Abstract

The exact etiopathogenesis of Kawasaki disease (KD), the most common childhood vasculitis, remains unknown; however, an aberrant immune response, possibly triggered by an infectious or environmental agent in genetically predisposed children, is believed to be the underlying pathogenetic mechanism. Patients with inborn errors of immunity (IEI) are predisposed to infections that trigger immune dysregulation due to an imbalance in various arms of the immune system. KD may develop as a complication in both primary and secondary immunodeficiencies. KD may occur either at disease presentation or have a later onset in IEIs. These include X-linked agammaglobulinemia (XLA), selective IgA deficiency, transient hypogammaglobulinemia of infancy; Wiskott–Aldrich syndrome (WAS), hyper IgE syndrome (HIES); chronic granulomatous disease (CGD), innate and intrinsic immunity defects, and autoinflammatory diseases, including PFAPA. Hitherto, the association between KD and IEI is confined to specific case reports and case series and, thus, requires extensive research for a comprehensive understanding of the underlying pathophysiological mechanisms. IEIs may serve as excellent disease models that would open new insights into the disease pathogenesis of children affected with KD. The current review highlights this critical association between KD and IEI supported by published literature.

## 1. Introduction

Kawasaki disease (KD) is the most typical childhood medium vessel vasculitis that usually affects children below five years of age [1]. However, older children and adolescents may also be affected. KD has a particular predilection for coronary arteries leading to coronary artery abnormalities (CAAs). The typical presentation of KD includes fever, erythematous rash, conjunctival injection, mucocutaneous involvement, and lymphadenopathy. Timely diagnosis and treatment with high-dose intravenous immunoglobulin (IVIG) are required in these children to prevent the development of CAAs and myocardial infarction. Hitherto, even after more than five decades of the initial description of the disease, the diagnosis of KD is clinical and based upon a set of clinical criteria [2]. The etiology of KD is still evolving, and there are no specific etiopathogenic mechanisms. Although the exact pathophysiology of KD is still under research, it is believed to be secondary to an abnormal/aberrant immune response possibly triggered by an infectious or environmental agent in genetically predisposed individuals [3].

Inborn Errors of Immunity (IEIs) are a heterogeneous group of monogenic disorders characterized by defects in the development or function of the immune system. These disorders can result in immune deficiencies, autoimmunity, autoinflammatory disorders, bone marrow failure, allergy, malignancy, and immune dysregulation [4]. Patients with IEIs are predisposed to develop an infection that triggers immune dysregulation, autoimmunity, or autoinflammatory disorder due to an imbalance in various arms of the immune system, such as phagocytic function, lymphoid system, humoral responses, and others [5]. The pathogenesis of KD revolves around a dysregulated immune system with a cytokine cascade in the acute phase [6]. The association between KD and specific IEIs is limited to certain case reports and case series and, thus, requires extensive research for a comprehensive understanding. In the present review, we aim to explore the interplay of the immune system in the pathogenesis of KD based on the published literature on the association of KD with IEI.

## 2. Methods

In this manuscript, we have reviewed the association of IEIs in the context of KD to understand the pathophysiology of KD. We searched the published literature online using the terms Kawasaki disease, mucocutaneous lymph node syndrome, primary immunodeficiency diseases, and inborn errors of immunity in PubMed, Embase, and Google Scholar databases. After an extensive search, we could retrieve 25 research articles on this topic, including case reports and case series, for inclusion in the present review.

## 3. Kawasaki Disease in Inborn Errors of Immunity

While KD is an immune-mediated condition, it is generally regarded as a complex multifactorial disorder with genetic and environmental factors contributing to its development [6]. KD may develop as a complication of an immunodeficiency disorder [7]. Patients with certain clinical types of IEI have shown KD during the disease course or at presentation [7]. These include antibody deficiency disorders, combined immunodeficiency disorders (CIDs), phagocytic defects, intrinsic and innate immunity defects, and autoinflammatory disorders as discussed below **(**Table 1).

### 3.1. KD and Predominantly Antibody Deficiencies

KD has been described in patients with antibody deficiencies such as X-linked agammaglobulinemia (XLA), selective immunoglobulin A (IgA) deficiency, and transient hypogammaglobulinemia of infancy. The first case report of the association of XLA and KD was by Behniafard et al., where-in a 15-month-old male infant suspected to have XLA because of absent tonsils, pan-hypogammaglobulinemia, markedly reduced B cells, and a family history of a confirmed diagnosis of XLA in sibling and maternal uncle presented with fever for five days with a neck mass, peripheral edema, and severe thrombocytosis with negative infectious work-up, diagnosed as atypical KD and the symptoms abated with high dose IVIG [8]. He also developed peri-ungual desquamation in 3rd week of illness. Another case report of a 3-year-old boy by Malekzadeh et al., who had several admissions due to autoimmune/immune dysregulation manifestations such as polyarthritis, and macrophage activation syndrome (MAS), presented with features of incomplete KD, and required methylprednisolone and IVIG for treatment, and was later diagnosed at six years to be XLA [9]. Both these cases had developed KD before any severe infectious complications and had not been on maintenance IVIG therapy. However, the case description by Sharma et al. of a 6-year-old boy diagnosed as a case of XLA at three years of age with a history of recurrent infections on regular maintenance IVIG infusion presented with manifestations of incomplete KD had a possible infectious trigger because of concomitant pneumonia during the episode [10]. Rivas-Larrauri et al. reported an 8-month-old boy with incomplete KD manifesting as fever, cough, rash, hoarseness of voice, thrombocytosis, and left coronary artery ectasia with concomitant XLA diagnosed based on *Pseudomonas aeruginosa* and *rhinoviral* infections, absence of Bruton tyrosine kinase (BTK) expression and a family history of XLA [7]. He responded well to IVIG, cyclosporine, and antibiotics.

Nishikawa et al. reported a patient with complete KD at two years of age with concomitant selective IgA deficiency [11]. The patient underwent serum IgA level assessment before initiating IVIG therapy and had low IgA levels, normal immunoglobulin G (IgG) levels, and high anti-IgG antibody titers, with low IgA levels in the sister. His symptoms resolved with the initiation of methylprednisolone [11]. Anzai et al. successfully treated a 5-year-old male with complete KD with intravenous cyclosporine A instead of IVIG who was diagnosed with selective IgA deficiency at the time of work-up for KD [12]. Şanlıdağ et al. presented a 4-year-old boy diagnosed with transient hypogammaglobulinemia of infancy at 3.5 years of age who developed incomplete KD [13]. He was treated with IVIG and aspirin and showed a good response.

### 3.2. KD and Immunodeficiency Disorders Affecting Cellular and Humoral Immunity

Among combined immunodeficiency disorders, an association between Wiskott–Aldrich Syndrome (WAS) and Hyper IgE syndrome (HIES), and KD has been reported. 

WAS is a combined immune deficiency characterized by eczema, thrombocytopenia, and immune deficiency. Autoimmune manifestations have been reported in 25–70% of children with WAS, with vasculitis being the second most common manifestation found in up to 30% of patients with WAS [27]. Large vessel vasculitis resembling Takayasu arteritis has been very well described with WAS in the literature, besides small vessel vasculitis like IgA vasculitis [14]. Kawakami et al. described a male infant with a typical triad of WAS carrying an intron six variant in *WAS* gene, who presented with classical features of KD in the form of fever, rash, dorsal edema, conjunctival injection, strawberry tongue, and cervical lymphadenopathy at six months of age [14]. His platelet count increased transiently in the acute phase of KD and decreased after IVIG therapy. He postulated that the acute phase of KD is associated with increased interleukin-6 (IL-6) levels with a thrombopoietic effect and could have led to a transient increase in platelet count in WAS.

Hyper IgE syndrome (HIES) is a rare IEI characterized by a triad of recurrent staphylococcal skin and lung infections, eczema, and elevated immunoglobulin E (IgE) levels. Autoimmune manifestations have been described in HIES [28]. Lupus-like phenotype with predominant renal involvement has been reported in patients with signal transducer and activator of transcription-3 (*STAT-3*) mutated HIES with anti-double stranded deoxyribonucleic acid antibodies (anti-dsDNA) positivity [29]. Association of HIES with KD has been described by Kimata et al. in two children from the same family, including a 10-year-old boy and his elder brother at three years of age, both of whom had a history of recurrent staphylococcal infections [15]. It is important to note that *STAT-3* loss-of-function mutation is associated with connective tissue abnormalities, including vasculopathy involving medium-sized arteries, particularly cerebral and coronary arteries, in the form of coronary ectasias and aneurysms [30]. Ling et al. described two adult patients with HIES with coronary artery ectasia and aneurysms detected by angiography following myocardial infarction [16]. Yared et al. reported a 15-day-old neonate diagnosed with HIES with a family history of KD in the elder brother [17]. Whether autoimmune phenomena play a role or an undiagnosed episode of KD in childhood, or connective tissue abnormality, causing ectasias in such cases needs to be understood. It can be accepted that there is an overlap between the HIES and KD, such as association with microorganisms like *Staphylococcus aureus* and *Candida albicans*, dysfunctional T cells, and dysregulated regulatory T cells (Tregs). Both autosomal recessive forms of HIES due to dedicator of cytokinesis 8 (*DOCK-8*) and tyrosine-kinase 2 (*TYK-2*) mutations), and autosomal dominant HIES due to *STAT3* loss-of-function mutation may lead to vasculitis [16]. Young et al. investigated a 30-year-old with HIES and CAAs. They showed a deficiency of cluster of differentiation 4 (CD4) + T central memory (CD4 + TCM) cells and memory B cells with an expansion of CD4 + terminally differentiated effector memory cells expressing CD45RA (TEMRA) cells [18].

### 3.3. KD and Phagocytic Defects

Chronic granulomatous disease (CGD) is an IEI characterized by recurrent infections by *catalase*-positive organisms and granulomatous inflammation with visceral abscesses due to defective nicotinamide adenine dinucleotide phosphate (*NADPH*) *oxidase* required for oxidative burst within phagocytes. These patients have an increased frequency of autoimmunity such as inflammatory bowel disease, lupus, rheumatoid arthritis, and IgA nephropathy [31]. KD has also been reported in patients with CGD. Yamazaki et al. described a 1-year-old boy with CGD who presented with atypical KD and CAAs and was treated with IVIG and steroids [19]. The authors hypothesized that defective *NADPH oxidase* causes impaired apoptotic debris clearance leading to persistent antigenic stimulation and exaggerated immune response. The hyperactivated neutrophils release proinflammatory cytokines. *NADPH oxidase* deficient T-cells show skewed cytokine production, T helper type 1 (T1) immune response, granuloma formation, and autoimmunity. The overlapping features between KD and X-linked CGD include immune-mediated generalized vasculitis and *Staphylococcus aureus* and *Candida* infections [19]. Muneuchi et al. described a 2-year-old male child diagnosed with X-linked CGD at four months of age due to glycoprotein 91 phagocyte oxidase (*gp91-phox*) gene mutation who presented with incomplete KD [20]. Tsuge et al. showed a 10-month-old male infant diagnosed with *gp91-phox*-mutated X-linked CGD at eight months of age who had complete KD [21]. Hule et al. reported two siblings from the same family who had an autosomal recessive form of CGD due to neutrophil cytosolic factor 1 (*NCF1*) gene mutation and developed KD [22].

### 3.4. KD and Defects in Intrinsic and Innate Immunity

KD has been reported in children with innate immune defects, including Warts, Hypogammaglobulinemia, Infections, Myelokathexis (WHIM) syndrome, and Signal transducer and activator of transcription 2 (*STAT 2)* deficiency [23,24]. Ma et al. reported a case of WHIM syndrome due to gain-of-function C-X-C motif chemokine receptor 4 (*CXCR4*) gene mutation with a history of leukopenia, neutropenia, and low immunoglobulin levels at birth who developed incomplete KD at one year of age without CAAs with increase/normalization of the neutrophil count during the KD episode [23]. His symptoms associated with KD, including coronary artery dilatation, improved after high-dose IVIG; however, leukopenia and neutropenia due to underlying *CXCR4* mutation persisted. Hambleton et al. described a 5-year-old male with *STAT2* deficiency who developed disseminated vaccine strain measles and presented as KD six days post-vaccination at 18 months of life [24]. The child responded to supplemental oxygen and supportive therapy.

### 3.5. KD and Autoinflammatory Disorders

Broderick et al. reported four children aged 10–36 months diagnosed with KD and treated with IVIG [25]. Post-IVIG, each child developed recurrent fever episodes with pharyngitis, aphthous ulcers, cervical lymphadenopathy, ocular symptoms, abdominal pain, and rash that responded to steroid therapy. Based on the signs and symptoms and response to steroid therapy with afebrile periods in between, they were diagnosed with periodic fever, aphthous stomatitis, pharyngitis, and cervical adenitis (PFAPA) syndrome that responded to steroid therapy. It was suggested that genetic predisposition leading to dysregulated innate immune response could be responsible for the pathogenesis of KD in PFAPA. Ninomiya et al. reported a 2-year-old girl diagnosed with PFAPA at one year of age who developed KD [26].

## 4. Discussion

According to the published literature, we could retrieve 25 cases, including 22 children and three adults affected with different clinical subtypes of IEI and developed KD during their disease. These include predominantly antibody deficiencies comprising XLA (*n* = 4), selective IgA deficiency (*n* = 2), and transient hypogammaglobulinemia of infancy (*n* = 1); combined immunodeficiencies including WAS (*n* = 1), and HIES (*n* = 6); phagocyte defects including CGD (*n* = 4); innate immune defects including WHIM syndrome (*n* = 1), and STAT2 defect (*n* = 1); and autoinflammatory diseases including PFAPA (*n* = 5). Of these, complete KD was documented in four patients, while incomplete KD was documented in nine patients. CAAs were seen in 9/25 (36%) patients with KD and IEIs.

### 4.1. Pathogenetic Mechanisms of KD in XLA

In XLA, patients develop subclinical infections leading to chronic inflammation and immune dysregulation. In response to infectious organisms, the expression of major histocompatibility complex (MHC) antigens increases via molecular mimicry on antigen-presenting cells (APCs), leading to T helper type 2 (T2) immune response and production of cytokines, including interleukins (IL-4, IL-5, and IL-10). The immune system shifts towards an autoimmune phenotype with aberrant B-cell function [8]. Additionally, overactive toll-like receptor-9 (TLR-9) and nuclear factor-kappa B (NF-κB)-mediated immune stimulation leads to the production of natural immunoglobulin M (IgM) antibodies from innate B-1 cells and T1 type of immune responses. The impairment of Tregs could also explain this association of KD in XLA [8]. Autoimmune mechanisms that may play a role in the pathogenesis of KD in XLA include leaky antibody production, defective BTK signaling leading to abnormal myeloid maturation and aberrant toll-like receptor (TLR) signaling, and autoreactive T cells [10]. XLA has been associated with inflammatory diseases with elevated levels of tumor necrosis factor- α (TNF-α) and interleukins (IL-10, IL-6, and IL-1α) secreted by peripheral blood mononuclear cells (PBMCs). The activity of inflammasome, nucleotide-binding domain, leucine-rich–containing family, and pyrin domain–containing-3 (*NLRP3*) activity is impaired in XLA as BTK is a regulator of *NLRP3*. Some infections, such as *Pseudomonas aeruginosa*, are found to be a common feature of XLA and infection-triggered KD [7].

In KD, B cells increase, whereas natural killer (NK) and T cells decrease in the acute phase. Serum IgG levels are significantly lower than normal age-matched controls (pre-IVIG), which has been found to correlate with disease severity, including CAAs and IVIG resistance [32,33]. Reduced B cell receptor (BCR) repertoire and B cell diversity have been detected through next-generation sequencing (NGS) in acute KD than febrile controls and post-IVIG therapy in KD [34,35]. Genome-wide association studies (GWAS) revealed down-regulation with reduced expression of B-lymphoid tyrosine kinase (*BLK)* and B-cell lymphoma 2-like 11 (*BCL2L11*) genes and upregulation with enhanced expression of *CD40, Fc Gamma Receptor Iia* (*FCGR2A*) and immunoglobulin heavy chain variable region (*IGHV*) genes leading to impaired function, activation and development of B cells [36]. The fact that the incidence of KD is extremely low in infants below six months of age and it increases after that due to declining maternal IgG levels also points towards the role of B-cell development and function in the pathogenesis of KD [36] (Table 2).

**Figure 1:**
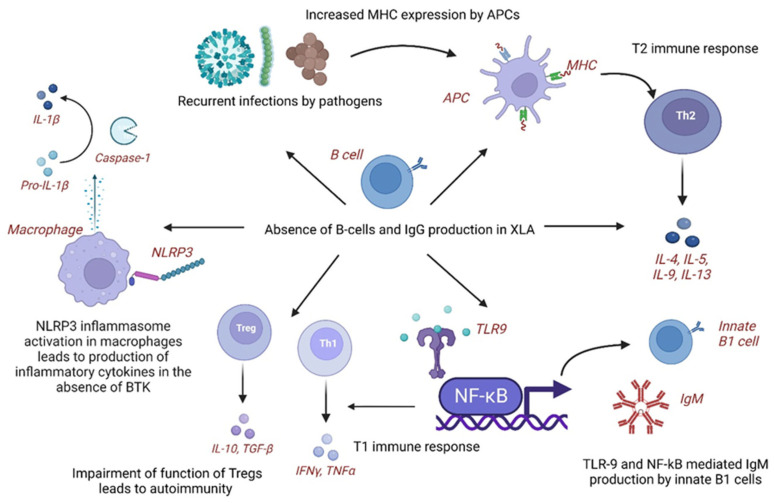
Cellular and molecular mechanisms underlying pathogenesis of Kawasaki disease in X-linked agammaglobulinemia. APCs: Antigen-presenting cells; BTK: Bruton tyrosine kinase; IgG: Immunoglobulin G; IgM: Immunoglobulin M; IL: interleukin; IFNγ: Interferon γ; KD: Kawasaki disease; MHC: Major histocompatibility complex; NLRP3: Nucleotide-binding domain, leucine-rich–containing family, pyrin domain–containing-3; NF-kB: Nuclear factor KB; T1: T helper type 1 immune response; T2: T helper type 2 (Th2) immune response; TLR-9: Toll-like receptor-9; Tregs: Regulatory T cells; TGF-β: Transforming growth factor-β; TNFα: Tumor necrosis factorα; Th1: T helper type 1 cells; Th2: T helper type 2 cells; XLA: X-linked agammaglobulinemia. Created with BioRender.com. https://www.biorender.com (accessed on 20 June 2023).

Thus, we hypothesize that XLA provides an excellent disease model for understanding the role of humoral immunity in the pathogenesis of KD. As B cell development and function are impaired in KD despite an increase in B cell number, this suggests that B-cell immune response plays a significant role in KD [36]. In XLA, the absence of B cells leading to severely reduced IgG levels may predispose these children to KD with CAAs, which needs further exploration (Figure 1).

### 4.2. Pathogenetic Mechanisms of KD in Selective IgA Deficiency

Autoimmune complications are reported in 7–36% of patients with selective IgA deficiency, including systemic lupus erythematosus (SLE), Sjogren’s syndrome, and juvenile idiopathic arthritis following sinopulmonary infections [11]. In patients with KD and concurrent selective IgA deficiency, IgM plasma cells might replace the function of IgA plasma cells that otherwise infiltrate coronary arteries and other tissues in KD. IgA plasma cells play an essential role in the inflammatory process associated with KD [12].

### 4.3. Pathogenetic Mechanisms of KD in Transient Hypogammaglobulinemia of Infancy

In transient hypogammaglobulinemia of infancy, incomplete KD could be due to partial humoral response due to hypogammaglobulinemia as B-cell-mediated antibody response plays a significant pathogenic role in complete KD [13].

### 4.4. Pathogenetic Mechanisms of KD in WAS

IL-6, a proinflammatory and thrombopoietic cytokine, is increased in the acute phase of KD [37]. Thrombocytopenia in WAS may respond to the stimulatory effects of IL-6 in acute KD. Other vasculitic disorders like Takayasu arteritis and IgA vasculitis have been reported in patients with WAS, indicating an increased predisposition to vasculitic diseases [14].

### 4.5. Pathogenetic Mechanisms of KD in HIES

**Figure 2:**
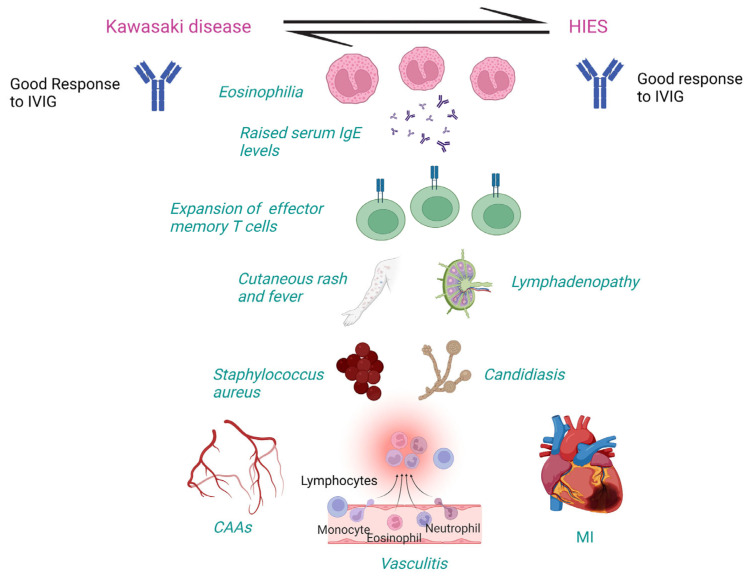
Clinical and laboratory features shared between Kawasaki disease and hyper IgE syndrome. CAAs: Coronary artery abnormalities, HIES: Hyper IgE syndrome; IgE: Immunoglobulin E; IVIG: Intravenous immunoglobulin; MI: Myocardial infarction. Created with BioRender.com https://www.biorender.com (accessed on 20 June 2023).

High-dose IVIG is an effective treatment for KD with HIES, HIES, and atopic dermatitis, as it significantly improves severe eczema. It reduces in-vivo and in-vitro IgE production from normal B-cells after stimulation with interleukin-4 (IL-4) and anti-CD40 antibody reagents [15]. Association between HIES and KD may exist due to the clinical overlap, especially fever, rash, and CAAs. Autosomal recessive (AR)-HIES patients may develop vasculitis, stenosis, and aneurysms [16]. AD or sporadic cases (*STAT-3* loss-of-function) may develop carotid and cerebral aneurysms secondary to connective tissue abnormalities. Thus, CAAs could be due to connective tissue abnormalities in HIES. Diagnosis of KD may be missed because of similar features between the two disorders [16]. KD and HIES share clinical features, including onset in childhood, CAAs, mucocutaneous involvement, lymphadenopathy, multiple genetic variations, and infection susceptibility (Table 3). AR-HIES (*DOCK-8* or *TYK-2* mutations) may also present as vasculitis like KD [17]. In KD, circulating CD4+ and CD8+ effector memory T cells with the proinflammatory phenotype and increased cytokine production have been detected in the acute phase with a rapid increase in the subacute phase followed by a decline in convalescent phase, indicating that antigenic exposure might occur days to weeks before the onset of acute phase [38]. HIES patients may show expanded terminally differentiated CD4+ effector memory T cells expressing CD45RA (TEMRA cells) associated with increased cytokine production leading to increased IgE levels, reduced CD4+ central memory T cells associated with decreased T-cell proliferation, and reduced memory B cells associated with impaired antibody responses in HIES [18]. CAAs could develop in HIES due to undiagnosed and untreated underlying KD [18]. Whether CAAs in patients with HIES are due to the primary disease or an episode of undiagnosed KD remains conjectural (Figure 2).

### 4.6. Pathogenetic Mechanisms of KD in CGD

**Figure 3:**
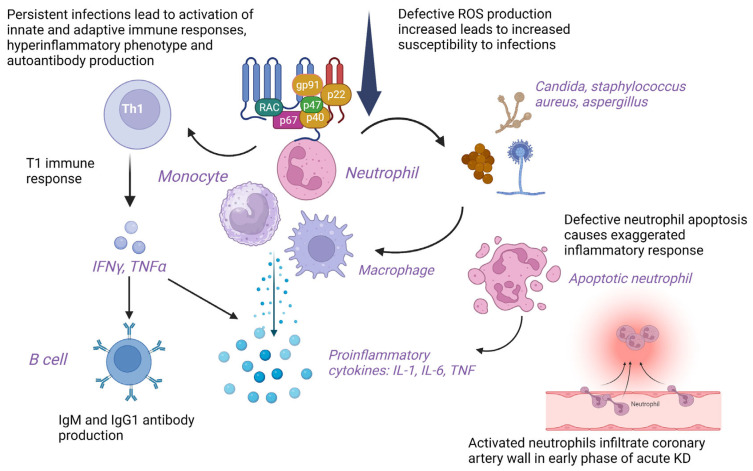
Cellular and molecular mechanisms underlying pathogenesis of Kawasaki disease in chronic granulomatous disease. gp91: 91-kilodalton phagocytic oxidase glycoprotein, IgM: Immunoglobulin M; IgG1: Immunoglobulin G subclass 1; IL: Interleukin; IFNγ: Interferon γ; KD: Kawasaki disease; p22: 22-kilodalton phagocytic oxidase protein (p22phox); p40: 40-kilodalton phagocytic oxidase protein (p40phox); p47: 47-kilodalton phagocytic oxidase protein (p47phox); p67: 67-kilodalton phagocytic oxidase protein (p67phox); T1 immune response: T helper type 1 immune response; Th1: T helper type 1 cells; TNF: Tumor necrosis factor; RAC: Rho-related C3 botulinum toxin substrate; ROS: Reactive oxygen species. Created with BioRender.com. https://www.biorender.com (accessed on 19 June 2023).

*NADPH-oxidase* deficient T cells in CGD show skewed T1 immune response with the production of T1 cytokines leading to autoimmunity [39]. Polyclonal hypergammaglobulinemia and autoantibody production are reported in patients with CGD and murine models, respectively [40]. Defective apoptosis of neutrophils and inefficient clearance of apoptotic debris in CGD may result in an exaggerated inflammatory response [40]. The ineffective removal of microbial pathogens and their antigens causes autoimmunity. *Candida albicans* and *Staphylococcus aureus* are common pathogens associated with KD and CGD [19]. Activated neutrophils infiltrate the coronary arteries with higher granulocyte-colony stimulating factor (G-CSF) levels detected in patients with CAAs than in patients without CAAs in the early phase of KD eliciting vasculitis due to mechanisms other than reactive oxygen species (ROS) generation. However, incomplete KD in CGD could be due to defective ROS production by neutrophils [19]. Predisposition to infections in CGD may delay the diagnosis of KD and cause vascular damage [20]. Insufficient ROS production in hyperactivated neutrophils and monocytes reduces immunoregulatory response leading to skewing of the immune system towards hyperinflammatory phenotype that may lead to KD. T cells are hyperactivated in CGD, evident by sustained elevation of soluble interleukin-2 receptor (sIL-2R) levels, leading to poor response to initial high-dose IVIG therapy [21]. Non-functional residual ROS generation leads to ineffective microbial clearance and persistent infection, triggering an abnormal immune response in AR-CGD. Activation of innate immunity and neutrophils with the release of proinflammatory cytokines such as interleukins (IL-1, IL-6) and TNF and activation of signaling pathways in CGD leads to an overwhelming immune response predisposing to KD [22] (Figure 3).

### 4.7. Pathogenetic Mechanisms of KD in WHIM Syndrome

Since the arterial endothelial cells regulate the expression of CXCR4, the up-regulation or down-regulation of CXCR4 or defective CXCR4/C-X-C motif chemokine ligand 12 (CXCL12) axis signaling can cause abnormal coronary artery development and endothelial cell dysfunction and endothelial injury leading to KD. Other autoimmune diseases reported in WHIM syndrome include diabetes and hypothyroidism [23]. Disseminated viral infection may mimic KD in patients with *STAT2* deficiency [24].

### 4.8. Pathogenetic Mechanisms of KD in PFAPA

Both KD and PFAPA may co-occur and are associated with activation of the innate immune system with similar cytokine profiles, including TNF-α and interleukins (IL1β and IL6). Levels of interleukin-1 receptor alpha (IL-1Rα), an inhibitor of IL-1β, are increased in response to IVIG [25]. The association between KD and PFAPA could be attributed to genetic predisposition leading to dysregulated innate immunity. NOD-like receptor (NLR) messenger RNA (mRNA) levels increase in patients with KD. NLR-ligand injection causes KD-like CAAs in mice [26].

### 4.9. Multisystem Inflammatory Syndrome in Children (MIS-C) and IEIs

Recently, a multisystem inflammatory syndrome in children, also known as MIS-C, has been identified as a rare but fatal complication of otherwise mild severe acute respiratory syndrome coronavirus 2 (SARS-CoV-2) infection in children and young adults [41]. It is characterized by a hyperinflammatory state that closely mimics KD. Unlike KD, which usually affects children below five years of age, MIS-C affects slightly older children aged between 7 to 12 years of age and has a more severe disease course with features of hemophagocytic lymphohistiocytosis (HLH) leading to multiorgan dysfunction syndrome (MODS), cytopenia, shock, and myocarditis [41,42,43]. Cytokine storm underlies the hyperinflammatory state. Thus, IEIs were hypothesized to be the underlying predisposing factors of this severe condition in affected children. Genetic defects, including defects in suppressor of cytokine signaling 1 *(SOCS1),* X-linked inhibitor of apoptosis *(XIAP)*, and cytochrome B-245 b-chain *(CYBB)* genes, have been identified in children with MIS-C using a targeted NGS approach. Autosomal recessive, bi-allelic loss-of-function or hypomorphic variants in 2’-5’-oligoadenylate synthetase 1 and 2 *(OAS1OAS2)* and ribonuclease L *(RNASEL)* genes involved in OAS-RNase L anti-viral signaling pathway have been identified in 5 children in a cohort of 558 children diagnosed with MIS-C using whole genome or exome sequencing approach by Lee et al. [44]. These children fulfilled the criteria of MIS-C and were recruited from 16 countries worldwide, with ages ranging from 3 months to 19 years and a male-to-female ratio of 1.5:1. In addition to these, variants in several genes implicated in IEIs, including HLH, such as perforin *(PRF1),* syntaxin binding protein 2 *(STXBP2),* unc-13 homolog D *(UNC13D),* lysosomal trafficking regulator *(LYST),* adaptor related protein complex three subunit Beta 1 *(AP3B1),* and dedicator of cytokinesis 8 *(DOCK8)* and genes involved in the interferon responses have been identified. However, the pathogenicity of these rare variants needs to be functionally validated. These studies suggest that children with IEIs involving the interferon response and regulatory genes and signaling pathways are predisposed to MIS-C [41]. Similar mechanisms might be operable in children with KD.

## 5. Future Directions

The significant interplay of the pathophysiological mechanisms between IEIs and KD is supported by the published literature. Although IEIs are considered rare disorders, they are being increasingly reported worldwide in different populations and ethnicities, having different clinical presentations, KD being one of them. Further research in this area may identify other types of IEI in KD patients. Targeted NGS panels, whole exome, and whole genome sequencing and transcriptome studies may unravel monogenic IEI defects in patients with KD; however, this may require larger patient cohorts. With recent advancements in precision medicine, such as targeted therapies and hematopoietic stem cell transplant (HSCT), the treatment of monogenic diseases has improved. Identification of monogenic defects in KD could provide newer insights into these patients’ pathophysiology, diagnosis, and management.

## 6. Conclusions

The reported data suggest that underlying IEIs are associated with an increased predisposition to the development of KD. Since IEI and KD have an early onset during childhood and share features attributed to immune deficiency and immune dysregulation with hyperinflammation, it would be interesting to investigate the patients with KD for monogenic IEIs. Thus, the significance of these case reports needs further evaluation and validation by analyzing larger patient cohorts.

## Figures and Tables

**Table 1:** Summary of clinical, laboratory findings, and treatment of KD in PID according to published literature.

Author (Year)	IEI	KD	Clinical Phenotype	Laboratory Findings	Treatment
*Predominantly antibody deficiencies*					
Behniafard et al. (2012) [8]	XLA at 10 months	Incomplete KD at 15 months of age	XLA: Absent tonsils, XLA in sibling and maternal uncle, KD: Fever for 5 days, right submandibular lymphadenopathy, peripheral edema, peri-ungual desquamation	XLA: Pan-hypogammaglobulinemia, markedly reduced B cells, BTK mutation confirmed in brother and maternal uncle. No infective organism identified KD: Anemia, thrombocytosis, elevated ESR and CRP, leukocytosis, hypoalbuminemia, gall bladder hydrops, ascites	IVIG (600 mg/kg followed by 2 g/kg) and antibiotics Asymptomatic on follow-up except for periungual desquamation
Malekzadeh et al. (2013) [9]	XLA at 6 years	Incomplete KD at 3 years	XLA: recurrent infections, JIA, and MAS, septicemia KD: Prolonged fever > 5 days, rash, cervical adenopathy, conjunctivitis	NA	Methylprednisolone and IVIG for MAS Methotrexate and prednisolone for polyarthritis
Sharma et al. (2017) [10]	XLA at 3 years	Incomplete KD at 6 years	XLA: Recurrent infections, CAP unresponsive to antibiotics KD: Prolonged fever, periungual desquamation, irritability	XLA: Hypogammaglobulinemia, absent B cells and BTK expression on flow cytometry, BTK gene exon 9 deletion, CXR showed consolidation, and PE KD: Anemia, thrombocytosis, elevated CRP, and ESR	IVIG (2 g/kg) and aspirin (50 mg/kg/day). Good response
Rivas-Larrauri et al. (2019) [7]	XLA at 8 months of age	Incomplete KD at 8 months	XLA: Infections, family H/O XLA in maternal uncles, one died in infancy due to sepsis and other in adulthood due to cancer KD: Fever, skin rash, cough, and hoarseness of voice	XLA: IgG and IgA were low (IgM: WNL), B cells 1%, reduced BTK expression on WB, *Pseudomonas aeruginosa* on blood culture, and *rhinovirus* positivity on PCR KD: Anemia with thrombocytosis, pericardial effusion, LCA aneurysm 3.4 mm (z-score > 4). Hypoalbuminemia, increased BNP	Good response to IVIG, corticosteroids, and antibiotics
Nishikawa et al. (2008) [11]	Selective IgA deficiency at 2 years	Complete KD at 2 years	Selective IgA deficiency: No specific infections KD: Persistent high-grade fever, conjunctivitis, diffuse skin rash, strawberry tongue, red cracked lips, peripheral edema, anorexia, and tachycardia	Selective IgA deficiency: Normal IgG levels but significantly low IgA levels (2 mg/dL), high anti-IgA antibodies, low IgA levels in sister KD: Leukocytosis, elevated ESR and CRP, hyperbilirubinemia, hyponatremia	Aspirin (30–50/kg per day) and urinastatin (15,000 to 25,000 U/kg per day, i.v. methylprednisolone 30 mg/kg/day for 3 days, followed by oral prednisolone (1 mg/kg/day). Symptoms resolved by 26th day of illness
Anzai et al. (2016) [12]	Selective IgA deficiency at 3 years	Complete KD at 5 years of age	IgA deficiency: RTI for 3–5 year and cellulitis post dental procedure KD: Persistent fever for 6 days, cervical LAD, conjunctival congestion, cracked tongue and lips and peripheral edema of hands	IgA deficiency: Low serum IgA level (<5.0 mg/dL) KD: Leukocytosis, elevated ESR and CRP, no CAAs on echo	Oral aspirin and i.v. urinastatin (fever persisted) i.v. CsA (3.0 mg/kg/day), then oral CsA (In remission)
Şanlıdağ et al. (2018) [13]	Transient hypogammaglobulinemia of infancy (3.5 years)	Incomplete KD at 4 years	THI: Recurrent tonsillitis and bronchopneumonia KD: Fever, strawberry tongue, conjunctivitis, and periungual desquamation	IgA deficiency: Low serum IgA level (<5.0 mg/dL) KD: Leukocytosis, elevated ESR and CRP, no CAAs on echo	Oral aspirin and i.v. urinastatin (fever persisted) i.v. CsA A (3.0 mg/kg/day), then oral CsA (In remission)
*Immunodeficiency disorders affecting cellular and humoral immunity*					
Kawakami et al. (2003) [14]	*WAS* (Early infancy)	Complete KD at 6 months	WAS: eczema, purpura, and recurrent infections KD: prolonged high-grade fever, skin rash, peripheral edema, cervical lymphadenopathy conjunctivitis, and strawberry tongue	WAS: microthrombocytopenia, *WASP* gene intron 6 597G→A missense mutation, confirmed by Western blotting Platelet count increased transiently in the acute phase of KD and decreased after IVIG	IVIG and oral prednisolone followed by high dose of IVIG (1 g/kg for 2 days and 0.5 g/kg for 2 days) (IVIG therapy may not increase platelet count in WAS)
Kimata et al. (1995) [15]	Pt 1: *HIES* at 1 year (male) Pt 2: *HIES* at 7 months (female)	Pt 1: KD at 3 years Pt 2: KD at 2.5 years	Pt. 1: HIES: Eczema, chronic otitis media, staphylococcal scalp abscess, pneumonia, pyothorax caused by *Staphylococcus aureus* KD: Fever	Serum IgE, anti-staphylococcal IgE levels, and in-vitro IgE production were increased pre-IVIG but significantly reduced post-IVIG treatment for 28 days	IVIG (400 mg/day for 28 days, following which fever, eczema, skin infections, and CAAs subsided dramatically in 6 months
Ling et al. (2007) [16]	HIES Pt 1: early childhood (male): sporadic Pt 2: 38 years of age (male): AD	Pt 1: CAAs at 43 years of age Pt 2: CAAs at 48 years of age	HIES: Pt 1: eczema, skin boils, recurrent aspergillus pneumonia, pneumatocele formation, coarse facies, scoliosis, and pathologic fractures CAAs: ventricular fibrillation (MI) Pt 2: recurrent pneumonia, recurrent sinusitis, boils, multiple fungal infections (histoplasmosis, cryptococcal meningitis, candidiasis), and coarse facies CAAs: Chest pain and tightness with dyspnea	Pt 1: HIES score: 79 (<40) CAA: fusiform aneurysm of 9 mm in left anterior descending and its first diagonal branch (mid-left anterior descending thrombus), diffuse ectasia in RCA on coronary angiography. Echocardiogram, CEMRI, and CT coronary angiogram: transmural infarction in the left anterior descending territory ECG and cardiac enzymes revealed acute anteroseptal MI Pt 2: HIES score: 79 (<40) ECG: Non-specific T-wave abnormalities Echocardiogram revealed left ventricular hypertrophy Coronary arteriography revealed showed RCA and proximal left anterior descending dilatation CT Coronary angiogram: 6.5 mm ectasia-aneurysm in left anterior descending and long RCA ectasia (6.5 mm)	Pt 1: Aspirin and Clopidogrel Pt 2: Aspirin
Yared et al. (2021) [17]	HIES (15 days after birth)	KD in 17-year-old elder brother at 3 years of age	HIES: Eczema, recurrent pneumonia with pneumatoceles, oral candidiasis, recurrent multiple site abscesses, sinusitis, toothache, cold mass in neck, pruritis, cough, left knee pain, coarse facies, joint hyperextensibility, and retained primary teeth	Serum IgE levels were elevated (2500 IU/mL), left submandibular abscess, with multiple lymphadenopathies Pus culture revealed methicillin-resistant Staphylococcus aureus	Antibiotics, antifungal, topical steroids
Young et al. (2007) [18]	HIES since childhood	CAAs at 29 years	HIES: Recurrent pneumonia, staphylococcal skin abscesses, pruritis, retained primary teeth, high arched palate, recurrent bacterial infections, hypertension, immune complex–mediated glomerulonephritis CAAs: substernal chest pain, elevated cardiac enzymes	Coronary angiogram revealed RCA aneurysmal dilatation with thrombus, left anterior descending, and circumflex arteries On flow cytometry, CD4+ TCM cells were reduced, whereas, CD4+ effector memory T cells and CD4+ TEMRAs were elevated	Fosinopril sodium, anti-coagulation, recanalization followed by cardiac stenting. Good response
*Phagocytic defects*					
Yamazaki-Nakashimada et al. (2008) [19]	CGD at 2 months of age (male)	Atypical KD at 1 year of age	CGD: Gastroenteritis leading to hypovolemic shock, pyelonephritis, pneumonia, and empyema. H/O parental consanguinity CGD in elder brother KD: Fever, cough, diffuse maculopapular rash, perineal erythema, BCG site ulcer, conjunctivitis, diffuse crackles, and hypertension post-therapy	CGD: NBT assay showed absence of NBT-positive neutrophils, CXR: Bilateral opacities in lungs KD: Anemia, leukocytosis with neutrophilia, thrombocytosis, elevated CRP, and ALT Echocardiogram: CAAs with dilatation of LCA (4 mm), and RCA (3.7 mm)	IVIG (2 g/kg), aspirin (60 mg/kg/day), Prednisone, Prazocin, and antibiotic On follow-up, asymptomatic with thrombocytosis, low Hb. RCA and LCA reduced in follow-up. Poor response to initial high-dose IVIG
Muneuchi et al. (2010) [20]	CGD, X-linked at 4 months of age (male)	Incomplete KD at 2 years	CGD: Disseminated and suppurative BCGosis KD: Fever, painful right cervical lymphadenopathy, conjunctival injection, red lips, BCG site reactivation	CGD: *gp91-phox* gene mutation <1% Rhodamine-positive neutrophils on DHR assay (70–100%) KD: Anemia, raised CRP, LCA dilatation with a maximum diameter of 4 mm on echo, followed by saccular dilatation with 6 mm diameter. Raised IL-6	Oral antibiotics and IFN-γ (subcutaneous), i.v. antibiotics, IVIG (2 g/kg and 1 g/kg/day for 2 days), aspirin, and dipyridamole. Poor response to initial high-dose IVIG. On follow-up improved
Tsuge et al. (2012) [21]	CGD at 8 months of age (male)	Complete KD at 10 months of age	CGD: perirectal abscess KD: Recurrent fever, maculopapular rash, peripheral edema, reddish lips, pharyngeal erythema, cervical adenopathy, HSM, and BCG site reactivation	CGD: Reduced superoxide production in neutrophils < 2% (70–100%), Frameshift mutation in *gp91phox* gene (exon 13, c. 1681 del G) KD: Anemia, neutrophilic leukocytosis, thrombocytosis, Raised CRP and AST, echo: WNL Raised IL-8 and sIL-2R levels even after IVIG for a few weeks	Perirectal abscess incision and drainage. High-dose IVIG (2 g/kg for 24 h, (1 g/kg/day for 12 h), flurbiprofen (5 mg/kg daily), antibiotics and antifungal therapy, aspirin (5 mg/kg/day). Poor response to initial high-dose IVIG
Hule et al. (2018) [22]	CGD, AR (p47phox−/−) (male)	Incomplete KD at 2 years of age	KD: Fever for 5 days, irritability, cough, erythema of palms and sole, and strawberry tongue CGD: Diagnosed on work-up after elder female sibling (7 years) was diagnosed with CGD	KD: Anemia, raised ESR and CRP, 2D echo: LCA aneurysm (3.5 mm, Z-score + 4.60) CGD: 0% NBT+ cells and 0% rhodamine+ cells (SI: 3.86) and deficient p47phox expression (S/N: 2.0). Homozygous missense mutation in *NCF1* gene, c.124C > T, exon 2, p. R42W	IVIG (2 g/kg/day) and aspirin. Good response
*Defects in Intrinsic and Innate Immunity*					
Ma et al. (2022) [23]	WHIM syndrome (neonatal onset)	Incomplete KD at 1 year of age	WHIM: neonatal jaundice, recurrent infections (H/O leukopenia, neutropenia, lymphopenia, Ig, and circulating B-cells in father) KD: Fever for 6 days, cough, erythematous rash on trunk, red lips, oropharyngeal erythema, tachycardia, conjunctival injection, cervical adenopathy	WHIM: Anemia, leukopenia with neutropenia during neonatal period/early infancy, low IgG and C4 level, CXR: bilateral pneumonia and infiltrates, BME: Hypercellular, mild granulocytic hyperplasia, maturation shift to right, with coarse cytoplasmic granules, and vacuoles, and nuclear multilobation. A novel AD heterozygous frameshift variant in the *CXCR4* gene (exon2, c. 1032_1033delTG, p. E345Vfs*12) in patient and father KD: Anemia, leukocytosis, elevated ESR and CRP, thrombocytosis. Echo: Short and thick LCA trunk, dilatation of left anterior descending artery (2.3 mm), and MR	No response to antibiotics. IVIG (2 g/kg), aspirin, and Dipyridamole (improvement in KD symptoms, including coronary artery dilatation but leukopenia and neutropenia continued to persist). IVIG and G-CSF were continued on follow-up
Hambleton et al. (2013) [24]	*STAT2* deficiency diagnosed at 5 years of age	KD at 18 months of age	*STAT2 deficiency:* disseminated measles (post-vaccination) KD: fever, rash, conjunctival injection, lymphadenopathy, hepatitis, and pneumonitis. H/O infant sibling death due to an overwhelming viral infection Parental consanguinity was present	Blood and BAL PCR revealed vaccine-strain measles virus. Absent STAT2 expression on immunoblot. DNA sequencing of *STAT2* gene revealed homozygous donor splice site mutation (introns 4 and 5, c. 381 + 5 G > C) in affected siblings (parents and other sibling were heterozygous) and 3 homozygotes from extended family. RT-PCR of cDNA revealed longer and less abundant products in patient than in control, indicating aberrant splicing and NMD	Child improved with supplemental oxygen and supportive care
*Autoinflammatory disorders*					
Broderick et al. (2010) [25]	PFAPA	KD (10, 12, 14, and 36 months of age), M: F-3:1	KD: Prolonged fever (7–35 days), conjunctival injection, erythematous rash, and lymphadenopathy (4/4), pharyngitis and strawberry tongue (3/4), and periungual desquamation and edema in 2/4, sterile pyuria in 1/4 cases. PFAPA: Recurrent febrile episodes, 2–24 months after IVIG, recurring every 2–6 weeks, lasting for maximum period of 10 days. Pharyngitis, and aphthous ulcers (3/4), lymphadenopathy (4/4), ocular symptoms (2/4), abdominal pain (1/4), and rash (2/4). All 4 patients were apparently well in between febrile episodes with normal CRP levels	Elevated CRP and ESR Echo revealed coronary artery dilatation in ¼ of patients	Responded to primary IVIG therapy (2 g/kg IVIg) followed by recurrent febrile episodes that responded to prednisolone in 3/3 cases. Tonsillectomy, in 1 case, improved the febrile episodes
Ninomiya et al. (2013) [26]	PFAPA at 1 year of age	KD at 2 years of age	PFAPA: Febrile episodes, cervical lymphadenopathy, tonsillar enlargement, and pharyngitis Fever persisted despite IVIG, possibly due to PFAPA but declined rapidly after prednisolone therapy KD: Fever, rash, cervical adenopathy, peripheral edema, and conjunctival injection	KD: Neutrophilic leukocytosis, raised CRP. No CAAs	Cimetidine (150 mg/day), IVIG (2 g/kg), Infliximab (high fever, mild tonsillar enlargement, mild cervical adenopathy persisted despite therapy) Prednisolone (2 mg/kg/day), following which fever subsided

Abbreviations: IEI: Inborn error of immunity; KD: Kawasaki disease; XLA: X- linked Agammaglobulinemia; BTK: Bruton tyrosine kinase; IVIG: intravenous immunoglobulin; ESR: erythrocyte sedimentation rate; CRP: C-reactive protein; JIA: juvenile idiopathic arthritis; MAS: Macrophage activation syndrome; NA: Not available; CAP: community-acquired pneumonia; PE: pleural effusion; H/O: history of; Ig: immunoglobulin; PCR: polymerase chain reaction; WNL: within normal limits; WB: western blotting; LCA: Left coronary artery; BNP: B-type natriuretic peptide; RTI: respiratory tract infections; CAAs: coronary artery abnormalities; echo: echocardiography; CsA: cyclosporine A; WAS: Wiskott–Aldrich Syndrome; WASP: WAS protein; HIES: Hyper IgE syndrome; AD: autosomal dominant; Pt: Patient; MI: myocardial infarction; RCA: right coronary artery; CEMRI: Contrast- Enhanced Magnetic Resonance Imaging; CT: computed tomography; ECG: Electrocardiography; IU: international units; CGD: chronic granulomatous disease; NBT: nitroblue tetrazolium test; CXR: chest X-ray; BCG: Bacille Calmette-Guerin; ALT: aspartate aminotransferase; Hb: Hemoglobin; gp91phox: 91 kilodalton phagocytic oxidase glycoprotein; DHR: dihydrorhodamine; IL-6: interleukin-6; IFN-γ: interferon- γ; HSM: hepatosplenomegaly; del: deletion; AST: aspartate transaminase; IL-8: interleukin-8; sIL-2R: soluble interleukin 2 receptor; AR: autosomal recessive; p47phox: 47 kilodalton phagocytic oxidase protein; SI: stimulation index; S/N: signal/noise ratio; NCF1: neutrophil cytosolic factor 1; C > T: cytosine > thymine; R42W: arginine 42 tryptophan; WHIM: Warts, Hypogammaglobulinemia, Infections, and Myelokathexis; C4: complement protein 4, BME: bone marrow examination; CXCR4: C-X-C motif chemokine receptor 4; E345V: glutamic acid 345 valine; fs: frameshift; 2D ECHO: 2-dimensional echocardiography; MR: mitral regurgitation; G-CSF: granulocyte colony stimulation factor; BAL: bronchioalveolar lavage; DNA: deoxyribonucleic acid; STAT-2: Signal transducer and activator of transcription; RT-PCR: reverse transcriptase PCR; cDNA: complementary deoxyribonucleic acid; NMD: nonsense-mediated mRNA decay; PFAPA: periodic fever, aphthous stomatitis, pharyngitis and cervical adenitis; M:F: male-to-female ratio.

**Table 2:** B-cell mediated humoral immunity in KD and pathogenetic mechanisms involved in KD associated with XLA.

KD	XLA with KD
Maternal IgG (humoral immunity) may be protective as incidence of KD is relatively low below 6 months of age and increases thereafter as the maternal IgG declines	Complete absence of B-cells and IgG in children with XLA may predispose to development of KD
Low IgG levels in acute KD have been associated with increased disease severity, CAA, and IVIG resistance	Recurrent or subclinical infections lead to chronic antigen stimulation and immune dysregulation, thereby, inflammatory response of KD. Certain infections like *Pseudomonas aeruginosa* are common between KD and XLA
B cells are increased in proportion compared to T and NK cells in acute KD	Elevated NLRP3 inflammasome activity and proinflammatory cytokines have been reported in XLA as loss of BTK leads to loss of negative regulation of NLRP3 activity
In acute KD, BCR repertoire diversity is reduced. B-cell-mediated immune responses are impaired	Increased MHC expression on APCs via molecular mimicry shifts the immune system towards T2 immune response with production of IL-4/5/10.
*BLK* and *BCL2L11* genes are downregulated and *CD40*, *FCGR2A*, and *IGHV* genes are upregulated	TLR-9 and NF-kB-mediated immune stimulation leads to IgM production from innate B1 cells and T1 immune response
Overall, B-cell function, activation, and development are impaired despite increase in number in acute KD	Leaky antibody production, defective BTK signaling leading to abnormal myeloid maturation and aberrant TLR signaling, autoreactive T cells, and impaired Tregs are other possible mechanisms causing immune dysregulation
Functionally impaired B-cells may predispose to KD	B-cell immunodeficiency with activation of non-B-cell-mediated autoimmune and proinflammatory pathways may cause immune dysregulation and predispose to KD in XLA

Abbreviations: APCs: Antigen-presenting cells; BCR: B cell receptor; *BCL2L11*: B-cell lymphoma 2-like 11; *BLK:* B-lymphoid tyrosine kinase; *BTK:* Bruton tyrosine kinase; CAA: Coronary artery abnormalities; *FCGR2A*: Fc Gamma Receptor IIa; *IGHV*: Immunoglobulin heavy chain variable region; IgG: Immunoglobulin G; IVIG: Intravenous immunoglobulin; KD: Kawasaki disease; MHC: Major histocompatibility complex; NLRP3: Nucleotide-binding domain, leucine-rich–containing family, pyrin domain–containing-3; NF-kB: Nuclear factor KB; T1 immune response: T helper type 1 (Th1) immune response; T2: T helper type 2 (Th2) immune response; TLR: Toll-like receptor; Tregs: Regulatory T cells; XLA: X-linked agammaglobulinemia.

**Table 3:** Clinical and laboratory features shared between KD and HIES.

Clinical and Laboratory Features	KD	HIES
Raised serum IgE and eosinophilia	✓	✓
Fever, rash, and mucocutaneous involvement	✓	✓
Susceptibility to *Staphylococcus aureus* and *Candida albicans*	✓	✓
Lymphadenopathy	✓	✓
Vasculitis, CAAs, MI	✓	✓
Response to IVIG with resolution of symptoms	✓	✓
Expansion of CD4+ effector memory T cells	✓	✓

Abbreviations: CAAs: Coronary artery abnormalities; HIES: Hyper IgE syndrome; IgE: Immunoglobulin E; IVIG: Intravenous immunoglobulin; KD: Kawasaki disease; MI: Myocardial infarction.

## Data Availability

Not applicable.
